# Evaluation of a protocol for resuscitation in burn patients

**DOI:** 10.1186/cc13881

**Published:** 2014-05-20

**Authors:** Manuel Sánchez-Sánchez, Abelardo Garcia-de-Lorenzo, Eva Herrero, Beatriz Galván, Maria J Asensio, Lucia Cachafeiro

**Affiliations:** 1 Unidad de Quemados Críticos, Servicio de Medicina Intensiva, Hospital Universitario La Paz/Idipaz, Paseo de la Castellana 264, 28046 Madrid, Spain

## 

We have read the commentary by Berger and Que [1] about our paper [2]. We are thankful to them for their comments; we would like to clarify some points.

First, the crude mortality rate, duration of mechanical ventilation, and renal dysfunction cannot be solely attributed to the resuscitation protocol. In addition, other factors are involved, such as intercurrent infection caused by an outbreak of multidrug-resistant *Klebsiella pneumoniae* strain [3].

Second, the relative initial elevation of the Sequential Organ Failure Assessment score or some intra-abdominal pressure measurements >20 mmHg cannot be interpreted as a failure due to resuscitation.

Third, clinical practices involving surgery, hydrotherapy under sedation, and other traumatic situations often prevent an adequate supply of nutrients with enteral nutrition; this problem is also applicable to those patients whose enteral nutrition is started early. Consequently, parenteral nutrition as a supplement to enteral nutrition is often recommended in such cases [4], which is actually not total parenteral nutrition, and the use of supplementary parenteral nutrition does not reflect gut dysfunction.

Finally, the use of a resuscitation protocol for monitoring transpulmonary thermodilution parameters at below-normal levels and ensuring tissue perfusion with lactate measurements was safe and avoided over-resuscitation, and was also necessary because hypovolemia might not be reflected by urine output. Future studies may reveal appropriate targets to achieve better results with this monitoring.

## Competing interests

The authors declare that they have no competing interests.
